# Calculating minimum safety distance against wildfires at the wildland-urban interface in Chile and Spain

**DOI:** 10.1016/j.heliyon.2022.e11238

**Published:** 2022-10-28

**Authors:** Miguel E. Castillo Soto, Juan R. Molina Martínez, Santiago Bonilla B, Roberto A. Moreno García

**Affiliations:** a Wildfire Laboratory, University of Chile, P.Box 9206, Santiago, Chile; b Department of Forest Engineering, University of Córdoba, Edificio Leonardo da Vinci, Campus de Rabanales, P.Box 14071, Córdoba, Spain; c Research Center for the Territory and Sustainable Habitat, Technological University of Indoamérica, Machala y Sabanilla, P.Box 170301, Quito, Ecuador; d Multidisciplinary Research Center of La Araucanía (CIMA), Autonomous University of Chile, P.Box 4780000, Chile

**Keywords:** Fire behavior, Radiant heat, Operational priorities, Self-protection measures, Setback distance

## Abstract

Wildfires in the urban-forest interface constitute a civil protection emergency, causing considerable personal injury and damage to properties. The potential impacts of wildfires on buildings can be minimized by reducing the surrounding fuel and the use of structural materials with low flammability. However, the costs associated with implementing these actions and the responsibility for maintenance usually present conflicts with the property owners. This study aimed to identify minimum safety distances in wildland-urban interfaces within priority areas. The priority areas were identified based on the integration of fire risk and fuel hazard. Radiant heat is a variable in the behavior of fire that directly influences the definition of safety distances. In this research the radiant heat transfer was calculated based on the potential fire behavior for each study area. A comparative study of the horizontal heat transfer method and the radiant heat flux model was carried out. The horizontal heat transfer method indicated the highest vegetation-free distances, ranging from 23 m to 32 m. Some safety distances were validated using experimental fires and wildfires. The findings from the experimental fires and wildfires emphasize the need for a progressive fuel load reduction to mitigate radiant heat transfer. This may include both the removal of surface fuel and removal of trees to mitigate against crown fires. Our findings provide relevant information for decision-making on the effectiveness and efficiency of safety distances at the wildland-urban interface.

## Highlights

• Radiant heat is a variable in the behavior of fire that directly influences the definition of safety distances.
• The safety distance against fires on depends of different slope scenarios.
• Forest fires and the forest urban interface are closely related through the definition of safety zones.
• The safety distance between houses and forest is a necessary criterion to consider for the defense against forest fires.

## 1 Introduction

Large fires are increasing in frequency on a global scale, especially due to climate change and the accumulation of available fuel to burn (Bowman et al., 2018; Rogers et al., 2020). Population growth and the growing demand for second homes or recreational homes in forest areas increase the vulnerability to forest fires (Reams et al., 2005; Castillo, 2006). When a forest fire spreads into the wildland-urban interface (WUI), people and property are likely to be impacted (Radeloff et al., 2005; Castillo and Garay, 2020). The WUI fire problem is not a new issue as evident by the recent wildfire incidents in Australia, the United States of America, Chile, Greece, and Portugal (Castellnou et al., 2018; Castillo et al., 2019; Gómez-González et al., 2018; Bento-Gonçalves and Vieira, 2020).

The economic losses from fires in the WUI may be very high, particularly in areas with a high density of houses (Román et al., 2013). Generally, the higher the fire intensity is associated with the higher likelihood of impact on buildings (Cohen, 2008; Castillo and Garay, 2020). The vulnerability of houses also depends on the vegetation surrounding the buildings and structural factors (Manzello, 2020; Pastor et al., 2020). The surrounding vegetation includes natural and ornamental vegetation as well as the horizontal and vertical continuity between them (Mell et al., 2010). The construction and decorative materials and the presence of hedges could impact the degree of damage (Ramsay et al., 1987; Manzello et al., 2017; Pastor et al., 2020). The distance between houses and the vegetation continuity is also of great importance in WUI impacts (Manzello, 2020). The settlements may not include protection systems against fire and embers. In many cases, the roads are narrow, winding, and without an escape route, as many settlements have no alternative access (Castillo, 2012; Manzello, 2020).

This WUI problem, with some exceptions, is similar in most Mediterranean countries, requiring building and planning regulations to mitigate fire impacts (Castillo, 2006). Garay et al. (2021) found possibilities to mitigate fire impacts based on building materials, the creation of vegetation-free distances and the reduction of the arrival time in the Chilean study area. In this sense, Chile and Spain have reviewed and adapted their current construction and fire protection standards in WUIs (Castillo et al., 2020; Pastor et al., 2020). Although many countries have dedicated efforts to the development of forest fire protection programs in WUIs (Castillo et al., 2012), there is a significant weakness in the creation and implementation of building and materials regulations (Koksal et al., 2019). To this end, many organizations and agencies are sharing responsibilities in civil protection against fire emergencies. However, it is not always possible to find effective coordination actions based on technical standards in fire defense areas.

Fuel management in the WUI requires the incorporation of safety distance or setback distance. Safety distance is the distance from the edge of the vegetation to a building or other inhabited area. This concept has been widely used to identify firefighters' safety distances in different fire scenarios (Butler, 2014; Page and Butler, 2017). Setback distance or home ignition zone (Cohen, 2004) has also been included by fire management regulations in some countries (Pastor et al., 2020). At present, fire regulation has only been tested in Australia (Australian Standard AS 3959-2009, 2010), although it should undergo modifications (Catalina, 2014), given the severe wildfires that have occurred in recent years.

Empirical or semi-empirical models of fire behavior (Byram, 1959; Rothermel, 1972; Finney, 1998; Castillo, 2013) have been widely used by fire simulators. However, there is great uncertainty about their validity for all ecosystems (Cruz and Alexander, 2013), particularly in the WUI (Penney and Richardson, 2019). At present, the fire simulation in the WUI are typically physical models of heat fluxes by radiation and convection (Muñoz and Navarro, 2020). The calculation of the radiation flux received by a house or other structure can be performed using the radiant heat flux model. The radiant heat flux model was based on the geometry of the flame (length and angle) has been gaining ground in recent years (Sullivan et al., 2003; Rossi et al., 2011). Some studies (Muñoz and Pastor, 2020) did not consider the convective flux in the WUI because it has a very small transfer in relation to the radiation flux. Morvan and Dupuy (2004) indicate that radiant and convective fluxes can be of the same magnitude in wind-driven fires. Other studies (Morandini and Silvani, 2010) demonstrated that the magnitude of radiant and convection fluxes depends on the fuel model and the wind speed. Finney et al. (2015) discussed the importance of convective heat for extreme behavior in real wildfires. There are several computational fluid dynamics (CFD) simulators, although today, most of them still require large computer and temporal resources to incorporate vegetation in a realistic way. The most widely used simulator for WUI studies is Wildland-urban Interface Fire Dynamics Simulation (WFDS) (Mell et al., 2010), FIRESTAR (Morvan and Dupuy, 2004) and FIRETEC (Linn et al., 2012).

This research aims to identify minimum safety distances in the WUI of two Mediterranean areas (Chile and Spain) using the radiant heat flux model. In the case of Chile, this Mediterranean climate is directly influenced by the Pacific Ocean, causing very marked thermal differences between winter and summer. Although vegetation-free strips are recommended in all WUI homes and buildings, these preventive actions against forest fires should be strengthened with additional fuel treatments, mainly in “very high” priority areas. This paper proposed a framework to operational priorities identification with a great adjustment in relation to fire-damaged homes in three real wildfires. The novelty of this research is the test of some safety distances based on thirteen wildfires and nine experimental fires. The identification of setback distances can assist in decision-making by ensuring the appropriate vegetation-free strip and additional fuel load strips, particularly in zones identified as priority areas. The minimum crown spacing for the prevention of crowing wildfires (Alexander and Cruz, 2020) is also an important outcome of this study, emphasizing the silviculture recommended for mitigating crown fire spread. Our findings provide new knowledge to improve the effectiveness of setback distances and tree crown distances reducing the impact of fires on people and their properties.

## 2 Materials and methods

### 2.1 Study area

**Figure 1:**
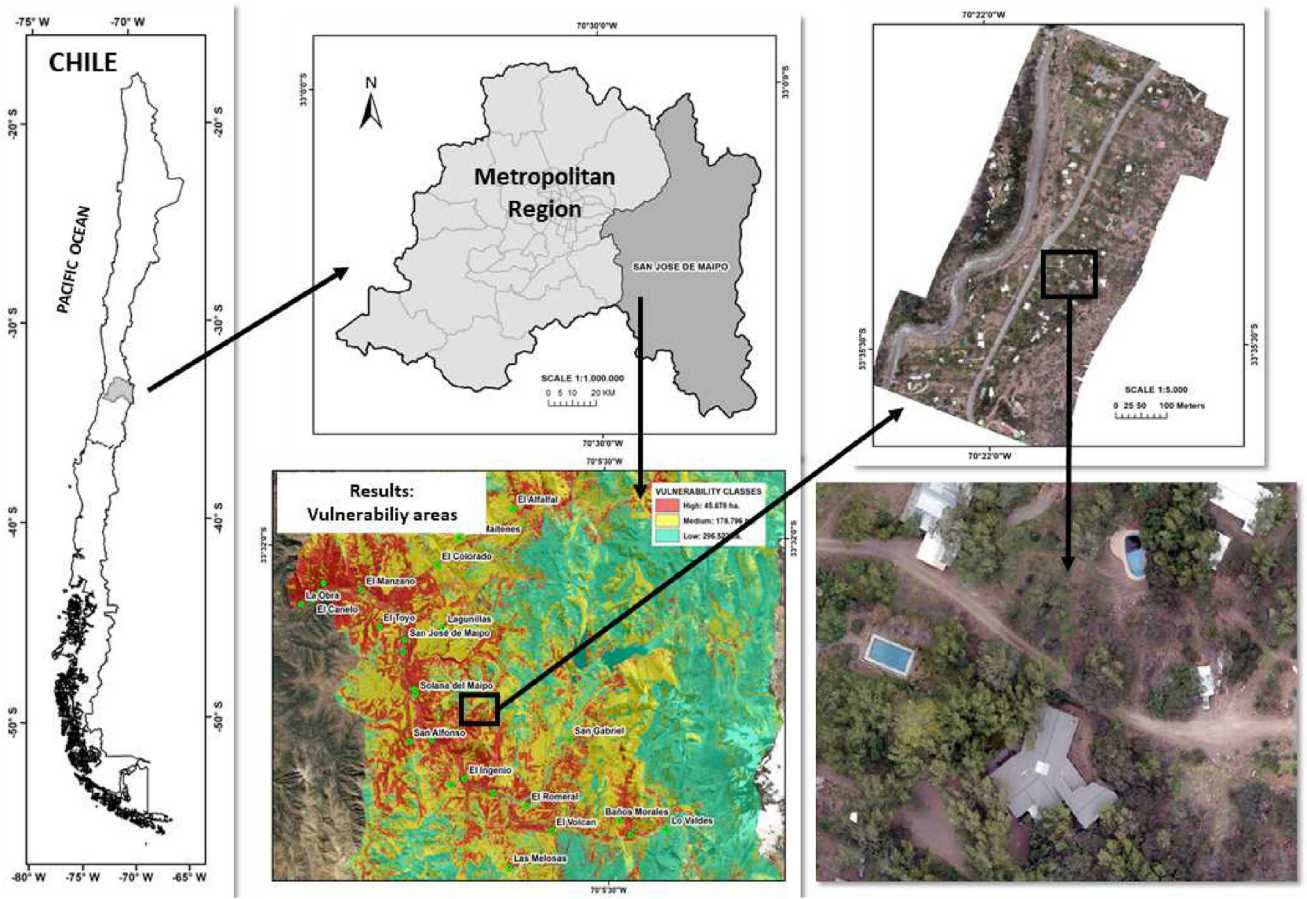
Operational priorities in the wildland urban interface in San José de Maipo (Metropolitan Region of Chile).

**Figure 2:**
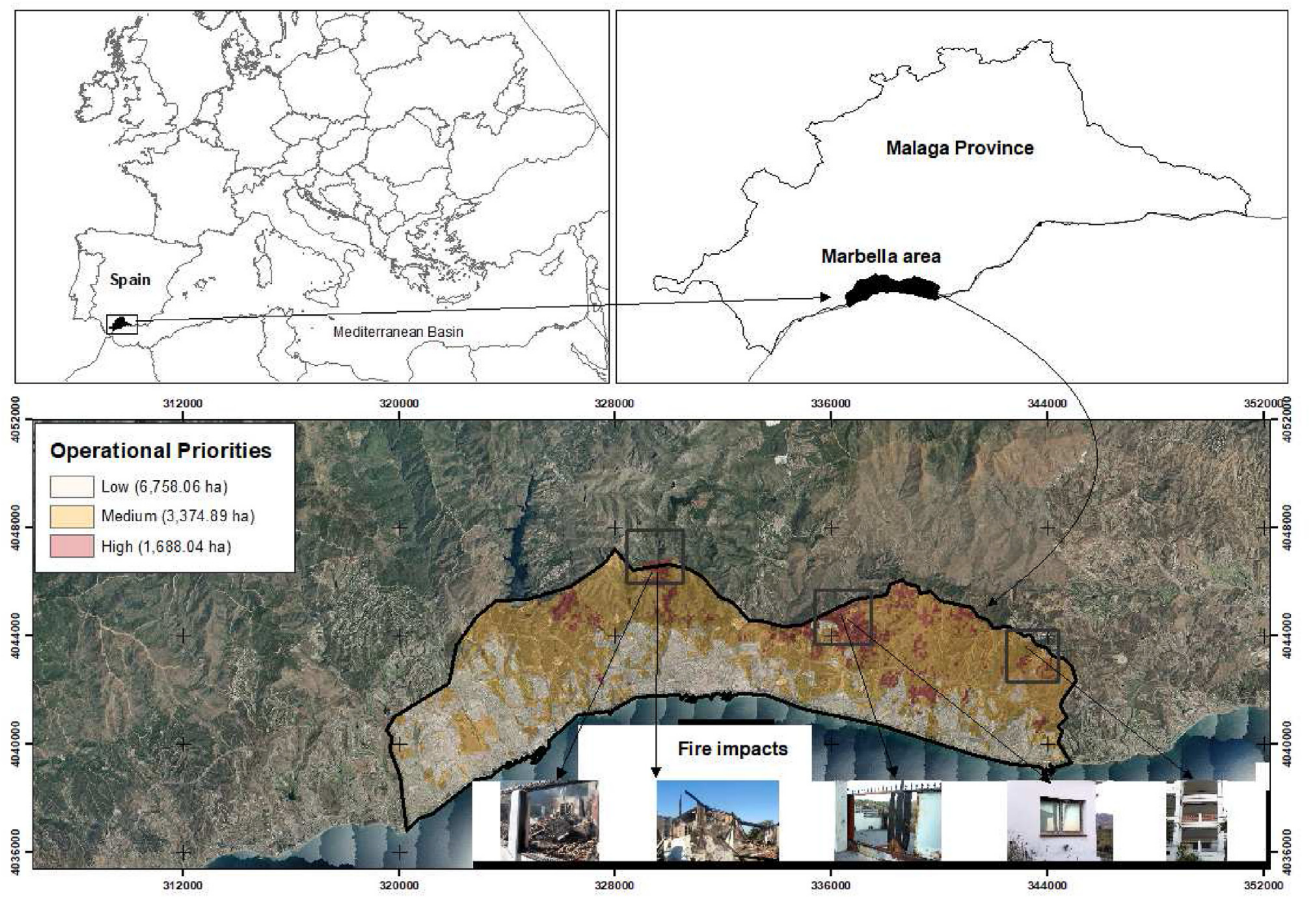
Operational priorities in the wildland urban interface in Marbella (Malaga Province in Spain).

This work was conducted in two areas with a Mediterranean climate and great wildfire risk in their WUIs. The first of these areas was located in the central area of Chile and the second in the south of Spain (Figures 1 and 2). These two countries were selected, given the differences in their fire regulations for the WUI. A comparative analysis of the two areas was carried out, both from an environmental point of view and from a regulatory point of view. The safety distances and the vegetation surrounding houses differ between the two study areas. Although there are previous tests that support these safety distances, these distances are binding according to national recommendations or regulations (Castillo and Garay, 2020; Pastor et al., 2020).

#### 2.1.1 Chile

For the period of 2015–2019, Chile has an average annual number of 6700 wildfires (>1 ha). The annual burned area has been highly variable due to the large fires that occurred in 2017 (Castillo et al., 2020b). The study area in Chile is located in the Commune of San José de Maipo in the Metropolitan Region of Chile, encompassing about 500,000 ha and 22 settlements. January and February are the highest fire risk periods with an average temperature of 28.1 °C and precipitation less than 10 mm. The climate of the study area is distinctly Mediterranean, with a very marked dry and warm period in summer and with scarce rainfall practically all year round (Castillo et al., 2020a). Therefore, these vegetation conditions lead to an increase the probability of large fire occurrence (Mouillot et al., 2002). Although there are different types of vegetation (Appendix I), dense shrubs and grasslands with trees dominate the study area, providing a highly flammable fuel.

In accordance with the Protocol of Forest Plantations in Chile (forest policy in Chile for 2015–2035), a free setback distance or strip width should be established based on the operational priorities of each area. Thus, the minimum width of the vegetation-free strip, measured in horizontal projection, would be 10 m for low priority, 15 m for medium priority, and 20 m for high priority areas. In addition to this vegetation-free strip, an additional surrounding strip over a longer width can be needed with a low fuel load and/or a horizontal and vertical vegetation discontinuity. Minimum widths for these additional strips would be 30 m for low priority, 55 m for medium priority, and 80 m for high priority areas. Periodic maintenance activities must be carried out. However, these fuel treatments usually present difficulties because they are not necessarily binding with a continuous landscape in which these protection strips are established as conglomerates. The latter is ongoing work to manage vegetation-free spaces on a more expanded landscape scale (Castillo and Garay, 2020).

#### 2.1.2 Spain

For the period of 2015–2019, Spain shows an average annual number of 3406 wildfires (>1 ha) and an average annual burned area of 92,591 ha. The study area is located in the municipality of Marbella (Málaga province), with a total of 12,000 ha and three urban areas. The climate is characterized by a lack of rainfall in the dry season and its annual irregularity. The mean summer temperature is over 30 °C, rising up to 40 °C in some years. Relative humidity drops with the Saharan-Africa winds known as “terrales”. The terrain has steep slope in the northern area, parallel to the sea, with many ravines and canyons. The ecosystems are dominated by shrublands (Appendix I), highly flammable and available to burn with summer conditions. There are also well-preserved forests of *Quercus suber* L. and *Pinus halepensis* Mill. forests.

The ornamental vegetation of the settlements increases the risk of home ignition. Three zones or strips are established surrounding the buildings based on national regulations (Decree 893/2013) and regional regulations (Law 5/1999, Decree 247/2001, Decree 371/2010). The first zone corresponds to a vegetation-free strip based on the surrounding slope. The minimum width of this strip is defined as 10 m (<20% of average slope) and the maximum of 35 m (70% of average slope). From this vegetation-free strip, a second strip or fuel treatment is established to create horizontal and vertical discontinuity of the vegetation. In this area, the distance between tree crowns and bushes should be more than 6 m. Therefore, the remnant trees must be pruned to a height of 2 m above ground according to current regulations. This second strip has a minimum width of 20 m (<20% average slope) and a maximum of 70 m (70% average slope). The last strip should have a distance between the tree crowns and between the bushes of 3 m. Similar to the second strip, in this third area the trees must also be pruned above 2 m and, regardless of the surrounding slope, must be at least 70 m. Consequently, the maximum recommended width of the fuel treatment corresponds to 175 m (35 m + 70 m + 70 m). Periodic maintenance activities must be carried out in all three zones.

### 2.2 Operational priorities for prevention and suppression of wildfires

Although standard safety distances could be used based on the national regulations of the study countries, a specific safety distance should be recommended for the areas of the highest priority. The operational priorities were identified according to the methodologies used and validated in each country (Castillo et al., 2013; Rodríguez y Silva et al., 2014). In both studied areas, the priority areas were established based on a multi-criteria analysis incorporating factors such as historical fire dataset, human pressure, meteorology, vegetation, topography, and resistance to control.

**Figure 3:**
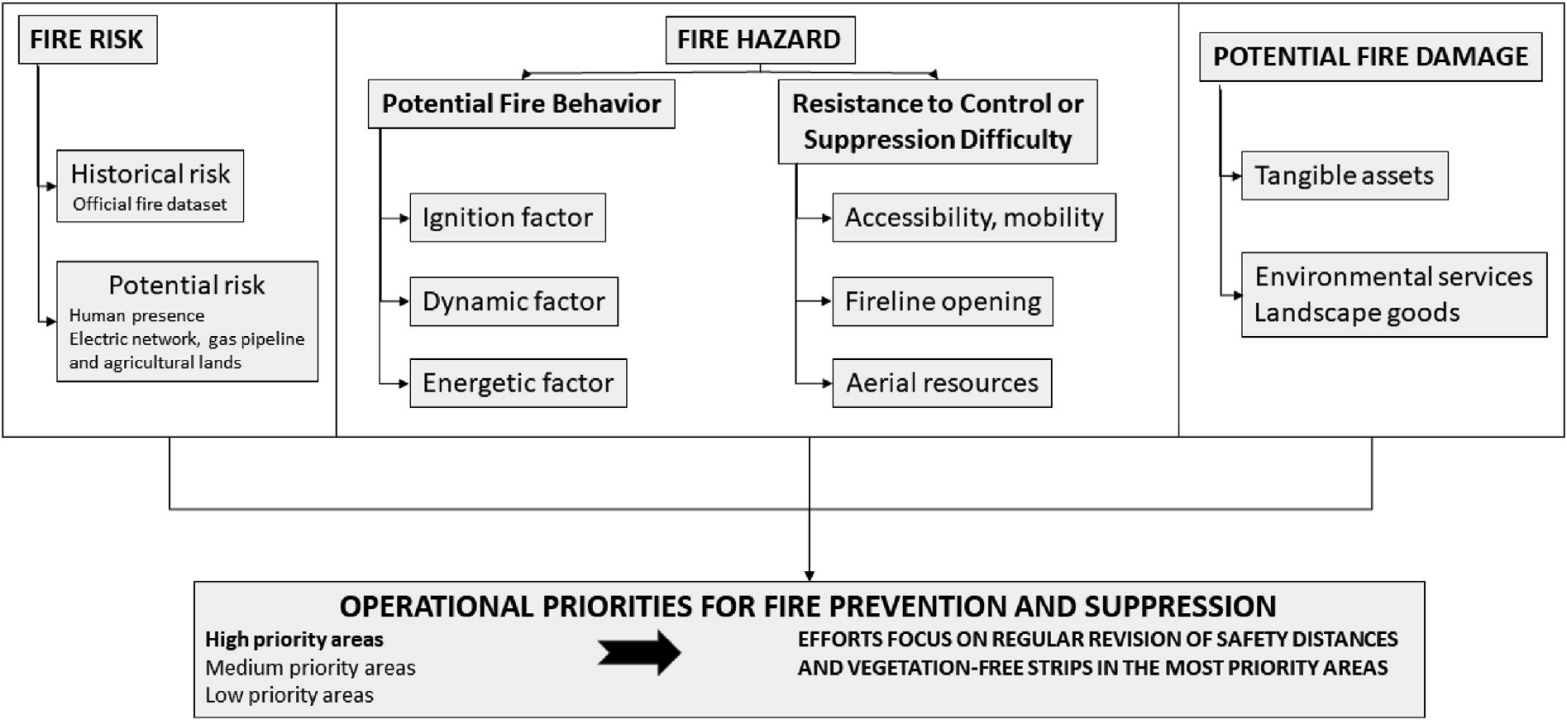
Framework for operational priorities identification.

The operational priorities framework was based on considering fire risk and fire hazard (Figure 3). Fire risk refers to the probability of ignition, whereas fire hazard is related to the degree of ease of fire ignition and spread (Hardy, 2005). In the case of Chile, potential damage was considered in the framework of the operational priorities according to the tested Chilean methodology (Castillo et al., 2013) (Figure 3). Cartographic information has been provided by the regional governments, including the wildfire dataset. The use of a Geographic Information System (GIS) allowed us to identify three priority categories (Low, Medium, High) with a spatial resolution of 25 × 25 m using the Jenks classification criteria described by Castillo et al. (2013). High priority areas would be associated with the need for greater safety distances or vegetation-free strips than the rest of the territory.

#### 2.2.1 Chile

The evaluation of the operational priorities was carried out through three general components: fire risk, fire hazard, and potential damage. Input variables (entrance data, source, and value range for each study area) are defined in Table 1. First, the fire risk included the historical fire risk (2005–2018 fire dataset) and the potential fire risk based on human pressure (settlements, roads and paths and tourist places). Historical and potential fire risk were calculated based on Castillo et al. (2016). Second, the potential fire behavior and the resistance to control were integrated into the fire hazard component. The calculation of the potential fire behavior required the following inputs: fuel model, fine dead fuel moisture content, live fuel moisture content, wind speed and direction, and topographic characteristics (Castillo et al., 2016). With these inputs, ignition, and dynamic and energetic factors could be generated by KITRAL model. In this sense, KITRAL model was used (Castillo et al., 2016) because it was developed, and validated comparing observed versus predicted spread rate for Chilean conditions. The resistance to control was established by each KITRAL fuel model according to fireline production rates (Castillo, 2013). Lastly, tangible assets, environmental services, and landscape goods were incorporated into the potential damage component (Castillo and Rodríguez y Silva, 2015a, Castillo and Rodríguez y Silva, 2015b). The Commune of San José de Maipo constitutes an area of high value for its environmental services and landscape goods, as well as the existence of valuable infrastructures.

**Table 1:** Variable data used to operational priorities identification.

Variable	Entrance data	Source	Chilean value	Spanish value
FIRE RISK				
**Historical risk**	Chilean fire dataset (2005–2018) and Spanish fire dataset (1991–2018)	Castillo et al. (2013) (Chile) and Rodríguez y Silva (2009) (Spain)	0–100	0–10
**Potential risk**			0–100	0–10
Human presence	Roads, paths and settlements cartography	Castillo et al. (2013) (Chile) and Rodríguez y Silva (2009) (Spain)	0–70	0–10
Electricity network, agricultural activities	Electricity and gas pipeline cartography and Corine land cover cartography	Rodríguez y Silva (2009) (Spain)	0–30	0–5
**FIRE HAZARD**				
**Potential fire behavior**			0–250	0–25
Ignition factor	Meteorological conditions (Appendix II) and topographic characteristics based on digital terrain model	Castillo et al. (2013) (Chile) and Rodríguez y Silva et al. (2014) (Spain)	0–80	0–5
Dynamic factor	Spread rate based on fire simulation using KITRAL (Chile) and UCO40 (Spain) fuel models	Castillo et al. (2013) (Chile) and Rodríguez y Silva et al. (2014) (Spain)	0–70	0–10
Energetic factor	Flame length or fire-line intensity and heat per unit area based on fire simulation using KITRAL (Chile) and UCO40 (Spain) fuel models	Castillo et al. (2013) (Chile) and Rodríguez y Silva et al. (2014) (Spain)	0–100	0–10
**Resistance to control or suppression difficulty**			0–250	0–1.43
Accessibility and mobility	Roads, ways and firebreak cartography	Rodríguez y Silva et al. (2014) (Spain)	-	0–20
Penetrability and fireline opening	Fuel model (KITRAL in Chile and UCO40 in Spain), soil cartography and digital terrain model	Castillo et al. (2013) (Chile) and Rodríguez y Silva et al. (2014) (Spain)	0–250	0–60
Aerial resources	Water sources cartography	Rodríguez y Silva et al. (2014) (Spain)	-	0–30
**POTENTIAL DAMAGE**				
Tangible assets	Land uses and settlements cartography	Castillo et al. (2013) (Chile)	0–150	-
Environmental services and landscape goods	Land uses, wetlands and lagoon cartography and digital terrain model	Castillo et al. (2013) (Chile)	0–150	-

The three components were converted to a common scale (0–100 points) through transforming maximum values to 100 points. A DELPHI method (De Villiers et al., 2005) was used to establish the relative importance of fire risk, fire hazard, and potential damage. Subsequently, the value set for each component was distributed among all the specific variables. The normalized score of each variable was also calculated using the DELPHI method, including government authorities and representatives of organizations and neighborhood associations of the study area.

#### 2.2.2 Spain

The evaluation of the operational priorities was carried out using two general components: fire risk and fire hazard. In this study area, potential damage was not included because the original methodology (Rodríguez y Silva et al., 2014) did not include this component, as well as the difficulty of testing in real fires. Input variables (entrance data, source, and value range for each study area) are defined in Table 1. First, fire risk considered the historical risk (1991–2018) and potential risk, equivalent to the Chilean methodology. For the historical risk (fire dataset), a non-parametric Kernel density analysis (Song, 1999) was performed using a search radius of 1000 m from ignition points. The maximum fire probability was in line with the maximum value given to this variable (Table 1). In the case of potential risk, the following activities were identified as risky based on the historical fire records: the human presence (Very High Risk), fireworks (High Risk), the electricity network, the gas pipeline, and the presence of agricultural activities (Moderate Risk). A buffer analysis of a 100-m area was carried out on roads and ways, settlements, fireworks show, electric networks, gas pipelines, and agricultural-forest interfaces. This distance threshold was selected due to its reliable results in other Mediterranean areas with similar characteristics (Zambon et al., 2019). “Very High” risk activities reached the maximum score (100%), whereas “High Risk” and “Moderate Risk” activities had 80% and 50% of the maximum score, respectively. If several activities were in the same pixel, the riskiest activity was considered. Second, the potential fire behavior and the resistance to control were integrated in the fire hazard component in a way similar to the Chilean methodology. Potential fire behavior was estimated using the BehavePlus modelling system (Rothermel, 1972; Andrews, 2014). The potential fire behavior was identified based on ignition, dynamic, and energetic sub-indices or factors (Rodríguez y Silva et al., 2014). The calculation of each of these sub-indices was carried out according to the methodology proposed by Rodríguez y Silva et al. (2014) with the UCO40 fuel modeling (Rodríguez y Silva and Molina, 2012) and meteorological conditions (Appendix II). The resistance to control was included by the methodology proposed by Rodríguez y Silva et al. (2014) according to suppression difficulty index. Therefore, the resistance to control also included other additional factors, such as accessibility, mobility, and aerial resources according to the previously tested methodology.

The importance given to the two general components and their associated factors were identified based on a DELPHI method, as in the Chilean methodology. DELPHI was carried out by three forestry engineers with knowledge of the study area and wildfire problem. One of the participants was a staff member of the local administration, another a member of the regional administration, and the third participant a university professor. Although methodological framework was validated in Chile and Spain using historical dataset (Rodríguez y Silva et al., 2014), the operational priorities cartography of the study area was tested using three WUI fires in the years 2012, 2019, and 2020. The first fire affected an area of more than 8000 ha, whereas the other two fires were smaller than 100 ha. The ratio of fire-damaged homes in “High Priority” areas with respect to the total fire-damaged homes was used to test the operational priorities cartography.

### 2.3 Estimation of the home safety distance

The calculation of the vegetation-free strip width to mitigate or avoid home impacts depends on a variety of factors, variables, and local conditions (Cohen and Butler, 1998), therefore, that a constant safety distance value may not respond to all local meteorological, topography, and fuel model conditions. Some practical implementations of this safety distance can also represent a significant outlay for small homeowners. Thus, the safety distance was calculated based on the potential fire scenario in each settlement (Massada et al., 2009). The weather conditions most likely to occur during the highest risk period were used to calculate the spread rate and heat transfer (see Appendix II). In this sense, temperature, relative humidity, wind speed, fine fuel moisture content, and slope (Appendix II) were used to generate fire behavior parameters (spread rate, flame length, and fire-line intensity) using Behave modelling system (Rothermel, 1972; Andrews, 2014) (Appendix II).

The safety distance of buildings depends on the meteorological conditions, but also that other factors such as the fuel model and the topographic characteristics were also decisive. As explained above, KITRAL fuel modeling and UCO40 fuel modeling were used for each study area. In both cases, these fuel models have been validated with field work. In the case of Chile, the validation process has been carried out consistently since 1999, with successive updates of the cartography. The major efforts were focused on hybrid fuel models (natural and ornamental vegetation with houses), which were not considered by previous fuel modeling classifications. In the case of Spain, the process was validated by a private company within the Local Planning for Forest Fire Emergencies (Marbella Local Government, 2018). Topographic characteristics were obtained using an official digital terrain model (5 m × 5 m of spatial resolution).

Some authors (Llinares, 2004; Catalina, 2014) pointed out that the maximum heat supported by the structural and decorative house materials ranges from 12.5 kW/m^2^ (wooden structures) to 400 kW/m^2^ (solid brick wall). For this study, the threshold or maximum heat supported by a building was established as 13 kW/m^2^. This threshold was justified by the presence of many houses with structures made of wood or decorative wooden elements. The technical building codes (EN, 1991-1-2) indicate fire damage to aluminum windows with heat transfer at around 13 kW/m^2^.

The heat per unit area depends on the heat of combustion, assuming 18,500 kJ/kg, and the available fuel load (fuel consumption ranged from 100% of the 1-h fuel timelag and 80% of the 10-h fuel timelag to 100% of the 1-h and 10-h fuel timelag, based on unfavorable meteorological conditions). The heat transfer is expressed as the heat released per area of the fire front in a given time. If the terrain slope is modified, the spread rate (Byram, 1959) and heat transfer will also be modified (1 kJ/s is similar to 1 kW). Thus, radiant heat transfer was calculated based on seven slope steepness thresholds (0%, 10%, 20%, 30%, 50%, 70%, and 90%), identifying the necessary safety distance for each of them.

Radiant heat transfer was calculated according to fire parameters and setback distance. The radiant heat flux model presents a wide versatility according to the fire behavior or flame characteristics and building environment. The properties of the flame and the characteristics of the mean of transmission (atmosphere) for each study area are shown in Appendix II. The model calculates the radiant flux (RHF) (Eqs. (1) and (2)) that falls on an object from the energy emitted by the fire front and that is not absorbed by the atmosphere (Eqs. (3) and (4)).

(1) RHF = *Ep* * τ * φ

(2) *Ep* = Ɛ * σ * *T*^4^

(3) *τ* = 2,02 * (P_w_ * x)^−0,09^

(4) P_w_ = P * Hr

where *RHF* is the radiant heat flux (kW/m^2^), *Ep* is the emissive power of the flame (kW/m^2^) calculated based on Eq. (2), *Ɛ* is the emissivity of the flame (it adopted a default value of 0.95 for wildfires, similar to Sullivan et al., 2003), σ is the Stefan-Boltzman constant (σ = 5.67 * 10^−8^ W/m^2^ *K^4^), T is the temperature of the flame (it adopted a default value of 1200 K similar to Sullivan et al., 2003), τ is the atmospheric transmissivity calculated based on Eq. (4), *P*_*w*_ is the partial vapor pressure of water (Pa) calculated based on Eq. (4), *P* is the vapor pressure of water under air temperature (it adopted a value of 4435 kPa for Chilean area and a value of 5522 kPa for Spanish area based on air temperatures in Appendix II), *H*_*r*_ is the relative humidity for each study area according to Appendix II, *x* is the effective distance between the flame and the building (m) and *φ* is the view factor that is calculated according to the equations proposed by Tan et al. (2005). The view factor (parameter to define the effects of orientation on radiation heat transfer between two surfaces) required the calculation of flame length and flame angle using KITRAL model (Chile) or Behave model (Spain). The RHF calculation was carried out in 6 slope classes, to determine the amount of energy emitted versus the distance in which the threshold of 13 kW/m^2^ is expressed.

### 2.4 Test of the home safety distance

#### 2.4.1 Width of the vegetation-free strip

There were many wooden houses or houses with decorative wooden elements in the housing structures, which usually ignite at 550–600 K (Albini and Reinhardt, 1995). Thus, we used the equivalence between house structure ignition and forest fuel ignition in the safety distance test. We analyzed the effectivity of fuel reduction or the setback distance to avoid ignition on the other side of the fuel reduction treatment. For this analysis, the fire front length played a key role in the dynamic and energetic progression of the fire (Anderson et al., 2015). While nine experimental fires allowed us to have accurate data about the heat flux with limited fire front length, thirteen wildfires made it possible to increase the analysis at longer fire fronts.

The width of the vegetation-free strip was tested by wildfires and experimental fires. Experimental fires (0.5–2 ha in size) were conducted for dense grasslands (model PCH3 in Chile and P7 in Spain), dense shrublands (model MT1 in Chile and M5 in Spain), and conifer litter with low woody fuel beneath a forest canopy (model HR5 in Spain). In the case of dense grasslands, three experimental fires and six wildfires were used to test fuel reduction treatments according to the fire front length. The range of the fire front and the vegetation-free strip was between 20–1000 m and 4–57 m, respectively (Molina and Rodríguez y Silva, 2017). The spread rate was estimated using thermocouple type K of 1 mm (experimental fires) and direct estimations with the help of the georeferenced position of the suppression resources (wildfires). While the spread rate ranged between 10–15 m/min and 32–35 m/min, the flame length was between 1–3 m and 2.5–5.7 m. In the case of dense shrubland models, three experimental fires and four wildfires were used to test the width of 10 m to avoid ignition on the other size. The spread rate ranged from 4.4 m/min to 30 m/min and the flame length was between 3.1 and 8.5 m. Lastly, the minimum safety distance of 5 m was tested for the litter fuel model using three experimental fires, with the spread rate ranging from 0.45 to 1.3 m/min and the flame length was between 0.4 and 0.8 m.

#### 2.4.2 Distance between tree crowns inside of the strips

All self-protection strategies of a structure from fire must be aimed at achieving a low-intensity surface fire. The distance between tree crowns plays an essential role in mitigating crown fire or intense fire and in reducing setback distance for house protection. Thus, the fuel treatment regulations for the WUI specify a minimum distance between trees to mitigate crown fire. A random stratified sampling using fire behavior (active crown, passive crown and surface fire) was used in treated areas and outside of them using circular plots of 15 m radius. Active crown, passive crown and surface fire behavior were identified by field observation based on crown fraction burned (Cruz et al., 2002). Fuel load was based on line transects and clipped vegetation plots (Rodríguez y Silva and Molina, 2012). Diameter at breast height, tree height, stand density, and crown diameter were identified in each plot. However, the variable of the greatest interest, given its influence on self-protection regulations, was the distance between tree crowns. Finally, fire behavior (flanking or heading fire) was considered in the field inventory.

The effects of crown distances were analyzed using two wildfires (years 2013 and 2019) with similar meteorological conditions of this study (Appendix II). These areas were treated from the edge of the vegetation-free strip before the fire with a width between 50 m and 100 m and different thinning intensities. The fuel models were timber (litter) in the treated areas and timber (litter and understory) outside of them. In the untreated area, the surface fuel load ranged from 13.75 t/ha to 42.11 t/ha and the shrub height was between 0.4 m and 1.5 m. The fine dead fuel moisture content was estimated between 6.5% and 8%, and the wind speed was between 10 km/h and 20 km/h. The fire behavior parameters were estimated by collecting information on the georeferenced location of the photos and videos. The spread rate prior to the preventive treatments varied between 4.7 m/min and 28 m/min and the flame length was between 8.5 m and 14.5 m.

## 3 Results

### 3.1 Operational priorities for prevention and suppression of wildfires

In the Chilean study area, the importance given to each component was as follows: 20% to fire risk (10% historical risk and 10% potential risk), 50% to fire hazard (25% potential fire behavior and 25% resistance to control), and 30% to potential damage according to DELPHI results. The “High Priority” areas covered almost 100% of the 22 settlements and 4000 buildings that were analyzed (Figure 1). The highest operational priority was 72 points out of 100 points. The buildings, which were considered based on the official land register, scored between 37 points (San José de Maipo Sur) and 60 points (El Melocotón). In flat areas and close to road intersections, building density was higher than in the rest of the study area. This house distribution was also associated with higher fire risk and fire hazard values.

In the Spanish study area, 154 settlements and 22,616 buildings were studied. The relative importance given to each general component was as follows according to DELPHI findings: 35% to fire risk (20% historical risk and 15% potential risk) and 65% to fire hazard (40% potential fire behavior and 25% resistance to control or suppression difficulty). The operational priorities were very heterogeneous, ranging from 4.2 to 78.99 points out of 100 points. There were small priorities in the southern area with smooth topography and close to the sea and high priorities in the mountain areas and those furthest away from the sea level (Figure 2). We could observe that 87.5% of the total fire-damaged homes were in the “High Priority” category according to the 2012 fire without fuel treatments. Other fire-damaged buildings, which were not included in this operational priorities' category, were affected by spotting on flammable material and not by direct radiation heat transfer. In the case of 2019 and 2020 fires, all fire-damaged homes were in the “High Priority” category.

### 3.2 Estimation of the home safety distance

**Figure 4:**
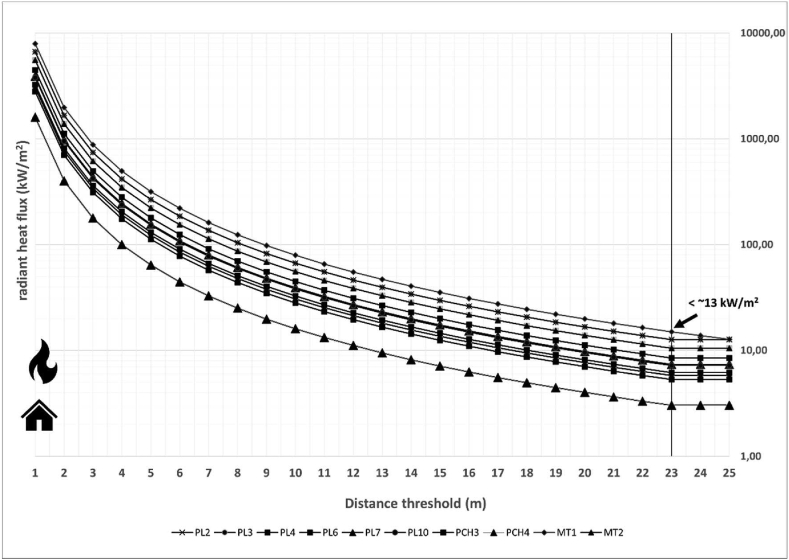
Estimation of radiant heat for Chilean fuel models in flat areas based on the distance from heat source. The threshold or maximum heat supported by a building was established as 13 kW/m^2^.

**Figure 5:**
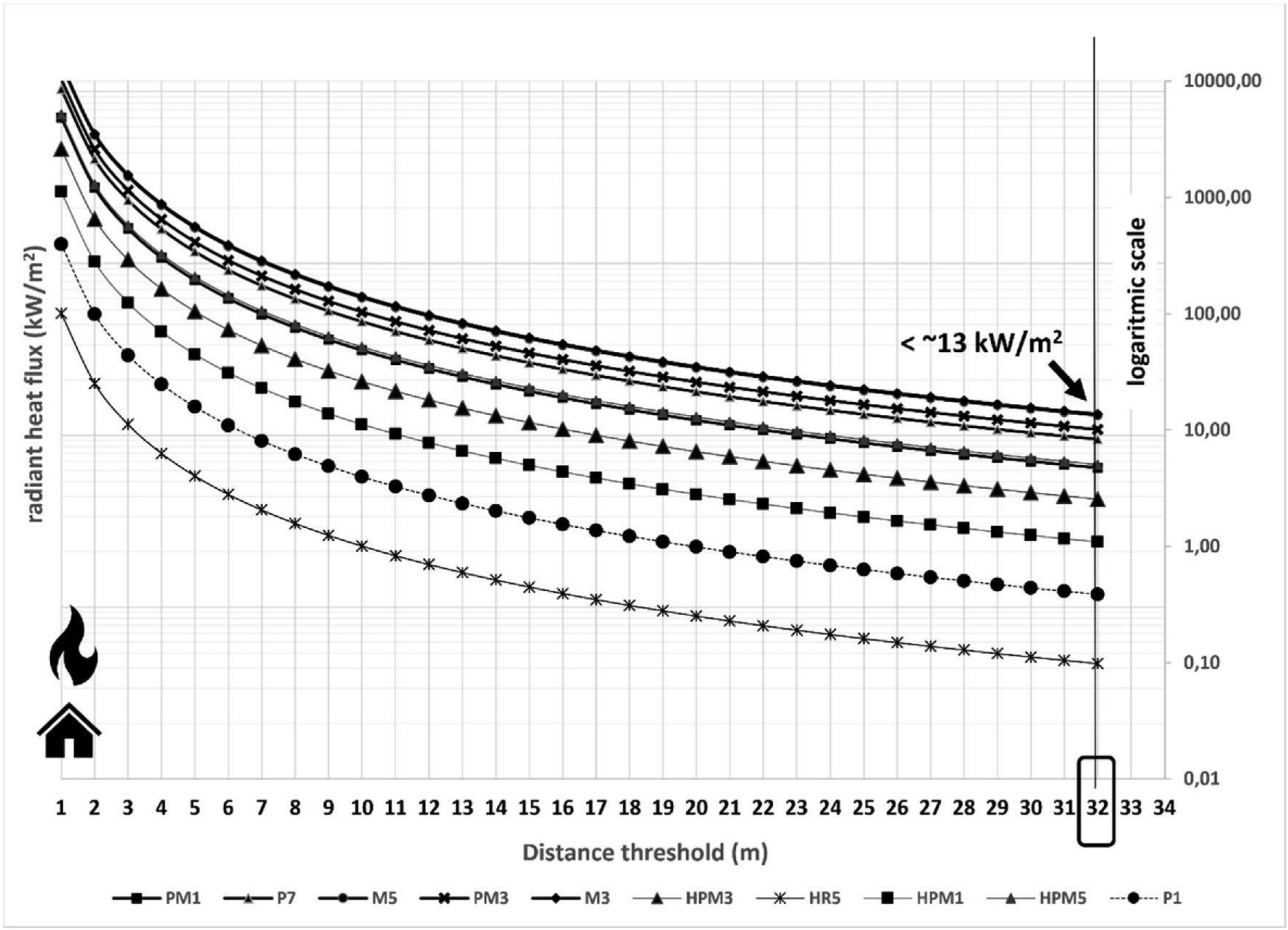
Estimation of radiant heat for Spanish fuel models in flat areas based on the distance from heat source. The threshold or maximum heat supported by a building was established as 13 kW/m^2^.

The flame length (flame length-fireline intensity relation according to Alexander and Cruz, 2012), the heat per unit area, and the radiant heat flux were modified based on the terrain slope and the fuel availability. In both study areas, the values of the spread rate and the fireline intensity exceeded 100 m/min and 15,000 kcal/m^/^s, respectively. In Chile, the radiant heat flux model resulted in safety distances between 8 m (PCH4 fuel model) and 20 m (MT1 fuel model) in flat areas (Table 2 and Figure 4). Large differences could be detected among the diverse Spanish fuel models ranging from 2 m (HR5) to 23 m (P7) in flat areas (Table 3 and Figure 5). The home safety distances were increased for many fuel models based on the slope of the surrounding terrain. In Chile, the safety distances increased with the slope reaching a maximum value of 23 m for shrublands (MT1) (Table 2). In Spain, the minimum safety distance ranged from 2 m (HR5 fuel model) to about 30 m (M5 and P7 fuel model) (Table 3). The highest safety distance was identified by P7 (32 m from slopes close to 90%).

**Table 2:** Safety distances (m) from heat source for Chilean fuel models using radiant heat flux model with a threshold of 13 kW/m^2^.

Slope (%)	MT1	MT2	PCH4	PCH3	PL2	PL3	PL4	PL6	PL7	PL10
10	20	20	8	14	20	18	15	18	17	17
20	20	20	8	15	20	18	16	19	17	18
30	21	20	10	15	21	19	17	19	18	18
50	22	21	11	16	22	19	17	19	19	20
70	23	21	12	16	22	19	18	21	20	21
90	23	21	14	17	22	20	19	21	20	22

**Table 3:** Safety distances (m) from heat source for Spanish fuel models using radiant heat flux model with a threshold of 13 kW/m^2^.

Slope (%)	P1	PM1	P7	M5	PM3	M3	HPM3	HR5	HPM1	HPM5
10	5	13	23	23	16	21	11	2	7	13
20	6	13	24	23	16	21	11	2	7	14
30	6	13	25	24	16	21	11	2	7	14
50	7	14	27	25	16	22	12	2	8	16
70	8	14	29	27	17	23	13	2	8	18
90	9	14	32	29	18	24	15	2	9	20

### 3.3 Test of the home safety distance

The safety distance for dense grasslands (PCH3 and P7) was established based on the fire front length (wildfires) or ignition line length (experimental fires) at the end of the vegetation-free zone. Safety distances between 7.5 and 11.5 m were needed to prevent radiation heat transfer with a fire front length lower than 100 m. Safety distances greater than 20 m were required by a fire front between 100 and 250 m. Larger wildfires with a fire front between 250 and 1000 m needed preventive treatments of 57 m to prevent radiation heat transfer. Nevertheless, in all studies of dense grasslands with fire fronts greater than 100 m, there were occurrences of fire spotting. A safety distance of 10 m for dense shrublands (MT1 and M5) was not effective in any of the three experimental fires. Fire ignition was produced beyond of the vegetation-free strip by heat radiation. Lastly, the safety distance of 5 m for the litter fuel model was effective for the three study experimental fires. Fire ignition was not produced beyond of the vegetation-free strip.

Analyzing stratified sampling (active crown fire, passive crown fire and surface fire), the tree crown distance ranged from 0 m (100% canopy cover) to 12 m. Active crown fire was found to crown distances lower than 1.5 m. Passive crown fire was identified at crown distances between 3(±0.6) m and 4.5 (±1.8) m. Only surface fire was observed by crown distances ranged from 7.5 m to 10.3 m.

## 4 Discussion

Wildfires are a civil protection emergency when they spread across settlements, causing risks to human life and properties (Radeloff et al., 2005; Cohen, 2008; Román et al., 2013; Castillo et al., 2019; Manzello, 2020). A more proactive approach is required in relation to fuel management in the surrounding home zone. The identification of optimal safety distances is a keystone to living in situations of confinement and fire defense (Reams et al., 2005; Pastor et al., 2020). This study suggests the optimization of safety distances using one physical model: radiant heat flux model (Sullivan et al., 2003). The recommendations of safety distances are directly related to the fuel model and terrain slope based on a potential fire scenario (Massada et al., 2009). The potential fire scenario was considered based on the weather conditions most likely to occur during the summer months. A new fire scenario and a higher radiant heat flux would need to be considered according to the further fire regime changes (Bento-Gonçalves and Vieira, 2020; Rogers et al., 2020).

This study proposes an integration scheme for operational priorities. The methodology was based on two types of factors: those associated with the fire probability and those related to the fire ignition and the fire spread. Further studies are needed to improve data integration and the relative importance of the inputs to extrapolate the operational methodology to any territory. The incorporation of suppression difficulty or resistance to control (Castillo et al., 2013; Rodríguez y Silva et al., 2014) is innovative in the mapping of the operational priorities. The author’s cartography showed a great adjustment of the real fire-damaged homes (2012, 2019 and 2020 fire events). Although vegetation-free strips are recommended in all WUI homes and buildings, fuel treatments should be strengthened with additional vegetation-free widths or low fuel strips in “very high” priority areas. In this sense, operational priorities cartography should be taken into consideration by fire managers to mitigate fire impacts on buildings and to make more effective making-decisions. All efforts must lead towards these priority areas which should have wider vegetation-free strips and should be treated previously.

Safety distance differences were found in the radiant heat flux model between Chile and Spain. Thus, the use of vegetation free strips was recommended at least in “High Priority” areas, which were identified in the cartography of the operational priorities. Thus, the differences were accentuated by the highest fuel loads and heat per unit area. Our safety distances are in line with standard distances previously established by different countries (Table 4). For example, Catalina (2014) noted that 90% of buildings with brush cleaning of 50 m did not have fire damage in France. However, some recommended safety distances (Nowicki, 2002) are higher than our findings. This difference could be associated with active crown fire that was not considered by our approach. We assumed a fuel treatment before the vegetation-free strip, so that fire would spread through understory in the surrounding home strips because of the fuel load reduction, the increase of the canopy base height and the high reduction of the canopy bulk density. Further studies should consider an assessment of crown fire behavior (flame length and angle) because our vegetation-free strips could be underestimated. Although some authors (Muñoz and Pastor, 2020) indicated a low relative importance of convective heat in WUI environments, other authors (Morvan and Dupuy, 2004; Morandini and Silvani, 2010) pointed out that convective heat could have importance based on the fuel model and meteorological conditions. Further studies should also include convective heat transfer of extreme fire (Finney et al., 2015) in the safety distances.

**Table 4:** Different recommendations about home safety distances around the world.

Zones	Source
Home-prevention zone between 5 m and 32 m based on fuel model and slope	Castillo and Molina-Martínez (2022) (in development)
Immediate Zone: (0–2 m around the house), Intermediate Zone (10 m around the house) and Extended Zone (20–30 m around the house) based on fuel model and slope	NFPA (2020)
Priority Zone 1 (0–10 m), Priority Zone 2 (10–30 m) and treated vegetation zone or Priority Zone 3 (30–100 m)	Partners in Protection (2003)
Home prevention zone (60 m)	Nowicki (2002)
Zone 1 (10–30 m) around the house, Zone 2 (10–30 m) and Zone 3 (to the property line) based on upon structure size and slope	Dennis (1994)
Home-prevention zone (30–60 m)	Cohen (2008)
Safety distance between 7–9 m and 26–30 m based on surface fire and fuel models	Zárate et al., 2008
A clean area of 5 m around houses and until 100 m of treated vegetation (8 times the vegetation height)	Pastor et al. (2020)
Until 40 m according to the flame size and the time of exposure	Cohen and Butler (1998)

Most standards or recommendations of the different countries indicate different vegetation-free strips around the buildings (Dennis, 1994; Cohen and Butler, 1998; NFPA, 2020; Nowicki 2002; Zárate et al., 2008; Pastor et al., 2020). Chile has no specific regulation and only considers technical recommendations (Castillo and Garay, 2020), showing a safety distance between 10 m and 30 m based on the surrounding area of the building. The Spanish regulation establishes 10 m of vegetation-free strip in the surrounding flat area of the buildings. Our findings, based on real data and experimental fires, indicated that a safety distance of 10 m is enough for P1, HR5, and HPM1, but the rest of fuel models require complementary fuel treatments. This result complies with the firebreak width that is necessary to stop grass fires based on fire intensity and the presence of trees (Wilson, 1988). For example, in dense shrubland burnings, the safety distance of 10 m was insufficient to prevent vegetation ignition on the other side of the vegetation-free strip. A vegetation-free strip on its own is unlikely to be sufficient to guarantee vegetation ignition on the other side of the strip. The temperature on the other side of the strip tested in this study was higher than 550 K (Albini and Reinhardt, 1995). Although the Spanish law increases the safety distance until 35 m according to the slope, a complementary fuel treatment or fuel load strip is necessary for the following Spanish fuel models: P7, M5, PM3, M3, and HPM5. The Spanish law establishes a second strip with a horizontal and vertical vegetation discontinuity ranging from 20 m to 70 m based on the terrain slope. Any fuel models around the buildings in this second strip should be modified to PM1 (treeless areas) or HPM1 (forest areas). In flat areas, even with this fuel model conversion, the safety distance would be even insufficient for P7, M5, PM3, M3, and HPM5. A third strip would be needed for P7, M5, PM3, M3, and HPM5. Therefore, when any of these fuel models are in “High Priority” areas, all buildings should fulfill the safety zones that are recommended by this study.

The silviculture recommended for second or third strips to mitigate potential fire spread depends on the tree crown distance (Alexander and Cruz, 2020). This variable is used more easily than tree distance, which would depend on the tree size. Thus, crown distance constitutes an important variable in the identification of technical criteria in different WUIs, mainly in PL (Chilean fuel models) and HPM and HR (Spanish fuel models). The crown distance should be at least 6 m in the second vegetation strip around houses according to Spanish law. Our findings on wildfire events add knowledge regarding thinning intensity or effective crown distance to mitigate crown fire spread. Active crown fire (Van Wagner, 1977) was not mitigated with tree crown distances less than 3.4 m. Information about crown distances in the range 4.5–7.5 m was lacking, but active fire spread did not stop with tree crown distances of 4.5 m. It was also important that the fires had flanking or heading behavior. In one analyzed fire, the two fire behaviors were identified, achieving different outcomes. A tree crown distance of 7.5 m for flanking behavior and a tree crown distance of 10.3 m for heading fire were necessary to stop crown fire. In all cases where crown fire was modified to surface fire, the canopy bulk density (considering only the needle foliage according to Alexander and Cruz, 2020) was around 0.08 kg/m^3^. The need for a minimum canopy bulk density to avoid crown fire spread has already been noted by Van Wagner (1977) and Alexander and Cruz (2020). Although canopy bulk density can be more consistent to identify crown fire behavior, crown distance is easier and cheaper variable to field inventories.

Vegetation-free strips involve large costs to private owners, particularly small homeowners (Manzello, 2020). The immediate home area or first vegetation-free distance should be treated annually to keep fuel loads to a minimum. To reduce costs to small homeowners, the second and third strips could be treated periodically to avoid hazardous fuel buildup according to vegetation regrowth situations. Our findings support the need for increasing fuel reduction measures as the distance to houses decreases in a similar way to existing approaches (Cohen and Butler, 1998; NFPA, 2020; Pastor et al., 2020). Tree removal and/or pruning are also required to mitigate crown fire spread in the surrounding areas of the settlements. A progressive increase in the tree crown distance could be a reliable approach to mitigate a surface fire spread.

One limitation of this study was the capacity of building ignition many kilometers from the fire head or source. While our study only looks at reducing the radiant heat influence, embers are responsible for many destroyed houses. Garay et al. (2021) explained the fire impacts due to the potential distance of embers, which exceeds the safety distances. This conclusion was reinforced in 2018 Spanish event, in which several reports have demonstrated that embers were transported via wind for more than 2 km, and still ignite a dwelling. Reducing the building vulnerability should include ember proofing properties (e.g.: ensure all sub-floors are enclosed, screens on doors and windows, no gaps around doors and windows) (Partners in Protection, 2003). In this sense, safety distance, vegetation-free strip, and tree crown distance recommendations should be complemented with further embers studies to reduce dwellings' vulnerability and to guarantee the safe confinement of the WUI population.

## 5 Conclusions

This research proposed a method to identify the optimal safety distance or vegetation-free strip, mainly in priority areas in the WUI. These priority areas were identified and tested by considering historical risk, potential risk, potential fire behavior, and resistance to control or suppression difficulty. Our methodological framework can be extrapolated to any WUI territory, independently of its spatial resolution and extension. The safety distance calculation was based on physical radiant heat transfer model according to the potential fire behavior of each area. The safety threshold was established as 13 kW/m^2^, based on the heat transfer need for the ignition of the most vulnerable building materials. Our recommended vegetation-free strips were between 5 m and 32 m for buildings due to their surrounding fuel models and terrain slopes. These recommendations differed from standard values used operationally without consideration to fuel model. Maximum recommended safety distances ranged from 23 m (Chilean area) to 32 m (Spanish area). However, some danger fuel models require additional strips or fuel treatments. Thus, tree crown distances are also recommended to mitigate crown fire around inhabited houses. The availability of safety distance tables allowed us to improve the effectiveness and efficiency in the design of home safety zones, reducing costs to small homeowners. Experimental fires and wildfires provided preliminary results for the effective calculation of the distances. However, further studies should increase the environmental conditions to test safety distances based on all fuel models and slopes.

## Declarations

### Author contribution statement

Castillo, M; Molina-Martínez, J: Conceived and designed the experiments; Performed the experiments; Analyzed and interpreted the data; Wrote the paper.

Bonilla, S; Moreno, R.: Analyzed and interpreted the data; Contributed reagents, materials, analysis tools or data.

### Funding statement

This work was supported by the CILIFO project (0753_CILIFO_5_E) from European Union (INTERREG VA Spain-Portugal).

### Data availability statement

Data included in article/supp. material/referenced in article.

### Declaration of interest’s statement

The authors declare no conflict of interest.

### Additional information

No additional information is available for this paper.

## Figures and Tables

**Table:** 

Vegetation characterization	KITRAL fuel model	Area (ha)	Relative area (%)
Immature conifer plantations (4–11 years), without forest management	PL2	324	0.48
Mature conifer plantations (12–17 years), without forest management	PL3	1258	1.85
Very mature conifer plantations (about 17 years), without forest management	PL4	3513	5.17
Mature conifer plantations (12–17 years), with forest management	PL6	76	0.11
Very mature conifer plantations (about 17 years), with forest management	PL7	61	0.09
Mature *Eucalyptus* spp. plantations (about 10 years)	PL10	12,124	17.85
Dense grasslands with herbaceous mesomorphic stratum or cropping lands	PCH3	14,381	21.17
Dispersed grasslands with herbaceous mesomorphic stratum or cropping lands	PCH4	8116	11.95
Dense shrubs with native mesomorphic scrubs	MT1	21,083	31.03
Medium density or dispersed shrubs with native mesomorphic scrubs	MT2	7004	10.30

**Table:** 

Vegetation characterization	UCO40 fuel model	Area (ha)	Relative area (%)
Short grasslands	P1	792	10.05
Mixture of grass and low shrub	PM1	760	9.64
Tall grasslands	P7	8	0.10
Dense and very high shrublands	M5	1373	17.42
Mixture of grass and dispersed high shrub	PM3	1972	25.02
Dense and high shrublands	M3	2338	29.66
Dense grass and/or shrubs mixed with litter from forest canopy	HPM3	98	1.24
High load conifer litter with light slash or mortality fuel	HR5	28	0.36
Low grass and/or shrubs mixed with litter from forest canopy	HPM1	84	1.07
Very dense grass and/or shrubs mixed with litter and down woody fuel beneath a forest canopy	HPM5	430	5.45

**Table:** 

Input	Chile (San José de Maipo)	Spain (Marbella)
Temperature (ºC)*	24.5–31.0	25–35
Relative air humidity (%)*	25–33	30–40
Wind speed 10 m above the ground (km/h)*	8–14.5	3.5–25
Fine dead fuel moisture content (%)*	11–13	5–8
Altitude (m)	625–970	0–1212.5
Slope (%)		
<10% (ha)	22,487,6	4209
10–20% (ha)	34,934,1	2515.5
20–30% (ha)	46,849,9	1610.5
30–50% (ha)	120,078	2263.4
50–70% (ha)	116,926	796.1
70–90% (ha)	52,426,3	264.25
>90% (ha)	105,292	66.56
Aspect		
North (ha)	99,306.1	1582.9
East (ha)	122,518	1492.4
South or flat (ha)	125,386	7145.1
West (ha)	151,783	1504.9
Fuel load (t/ha)	0–95	1.77 (P1) - 68.4 (M9)

**Table:** 

Intermediate variable	Chile (San José de Maipo)	Spain (Marbella)
Spread rate (m/min)	24.5–31.0	1.3–196.4
Flame length (m)	25–33	2.3–14.4
Heat emitted by fire or Q_0_ (kW)	0–95	332.7–93,041.74

## Appendix I Vegetation types and fuel models distribution.

I.1. Vegetation types and fuel models distribution for Chilean study area.

I.2. Vegetation types and fuel models distribution for Spanish study area.

## Appendix II Inputs used for the operational priorities' assessment.

II.1. Range of meteorological, topographic and fuel model inputs to operational priorities calculation.

*Meteorological conditions have been calculated based on the most likely conditions that occur in summer period (broad of the conditions in the middle of the days). While temperature is calculated as the mean of daily maximum temperatures, relative humidity and fine fuel moisture content is calculated as the mean of daily minimum values. Wind speed is calculated as the mean of a 30-minute dataset in the middle of the summer days.

II.2. Range of fire behavior parameters of the study areas.
